# The role of psychological distress, stigma and coping strategies on help-seeking intentions in a sample of Italian college students

**DOI:** 10.1186/s40359-023-01171-w

**Published:** 2023-06-06

**Authors:** Jessica Dagani, Chiara Buizza, Clarissa Ferrari, Alberto Ghilardi

**Affiliations:** 1 Department of Clinical and Experimental Sciences, Section of Clinical and Dynamic Psychology, University of Brescia, Viale Europa, 11, 25123 Brescia, Italy; 2 Service of Statistics, IRCCS Centro San Giovanni di Dio Fatebenefratelli, Via Pilastroni, 4, 25125 Brescia, Italy

**Keywords:** University students, Counselling, Help seeking behaviour, Psychological distress, Stigma, Coping strategies

## Abstract

**Background:**

Mental health issues are common among university students, but the latter are unlikely to seek professional help even when mental health services are available. Coping strategies, stigma and psychological distress are often considered as factors that can affect help-seeking intentions in university students.

**Methods:**

This study aimed to determine the role of coping strategies, stigma and psychological distress on the intentions to seek professional help for psychological problems. All students (N = 13,886) from an Italian medium-sized university were asked to participate in a multidimensional online survey and 3754 (27.1%) agreed to participate. A Structural Equation Modelling approach was applied to explore the simultaneous direct and indirect effects of distress, stigma and coping strategies on professional help-seeking intentions.

**Results:**

Results showed that students were not very likely to seek professional help and, through the Structural Equation Model, psychological distress was found to be positively correlated with coping strategies, which in turn was negatively associated with the stigma of seeking help. The latter was negatively associated with professional help-seeking intentions. These effects suggest that students with significant psychological distress use coping strategies to face the stigma of seeking help: the lower the stigma of seeking help, the higher the chance of developing intentions to seek professional help.

**Conclusions:**

This study suggests the importance of implementing programs to encourage college students to seek help, including measures that foster a stigma-free environment, reduce psychological distress and promote the use of adaptive coping strategies. Interventions should be focused firstly on self-stigma and secondly on perceived stigma, taking into consideration the level of psychological distress and social stereotypes associated with mental disorders and help seeking behaviours. Programs about coping are also essential and should focus on promoting emotion-focused strategies and problem-focused strategies.

**Supplementary Information:**

The online version contains supplementary material available at 10.1186/s40359-023-01171-w.

## Background

University years represent a challenging transition from childhood to adulthood and university itself is a highly stressful experience for students [1]. There is a significant number of stressors that students face every day and that contribute to the experienced stress, including financial pressures, changes in social support, adapting to a new routine, forming new relationships, and academic rigor [2, 3]. Indeed, most students report a high level of psychological distress [4], while mental health issues are not uncommon [5, 6]. However, research indicates that the student population is quite reluctant to seek professional help for mental health problems [7], with men having more negative attitudes than women towards help seeking [8, 9]. In a recent survey conducted by [10], results showed how people not presenting help seeking behaviour were more likely to have severe psychological distress, and this trend appeared to be slightly stronger among young people aged between 20 and 39. Data from research in college settings confirm these results, suggesting that students may tend not to seek help, even when mental health services are available [11].

Italian college students' situation is in line with the results reported by international studies: Porru and colleagues [12] found that 78.5% of the students participating in their study experienced psychological distress, while Bert and colleagues [13] found alarming rates of depressive symptoms, suicidal ideation and perceived stress (30.6%, 8.8% and 87.7% respectively). With regard to help-seeking attitudes, so far only a few Italian studies have been conducted. In their cross-sectional study including a large sample of Italian college students, Fiori Nastro and colleagues [14] found that only 22.3% of the students in need of psychological support asked for professional help. They also found that unwillingness to seek help was significantly associated with several factors including stigma, being male and distrust in existing mental health services. These results are quite similar to those obtained by Ebert and colleagues [15] in their international web surveys, which were part of the WHO World Mental Health International College Student Initiative. The authors included several colleges and universities in eight different countries (not including Italy), reporting that only one in four of the almost 14,000 participating students would seek treatment if they had a future emotional problem [15].

The ability to seek professional help for psychological issues is one form of individual coping [16], which is necessary when dealing with stress and accompanying stressors. Coping styles have been categorized into three dimensions: problem-focused, emotion-focused and dysfunctional or avoidance coping [17, 18]. Problem-focused coping includes the active efforts to deal with the source of stress via individual behaviour, while emotion focused-coping is when the person attempts to diminish the emotional consequences, and handle thoughts and feelings associated with the stressors. The use of both problem- and emotion-focused strategies was associated to reduced psychological stress [19, 20]. Talebi and colleagues [21] found that emotion-focused strategies contributed to both self and perceived stigma of help-seeking, whereas problem-focused strategies was related to decreased self-stigma. Avoidance or dysfunctional coping refers to strategies which avoid dealing with either the problem or the associated emotions, which in turn may play a role in preventing help seeking [22].

The use of effective coping strategies may be hindered by several factors, including stereotypes and stigma. Stigmatization of people with psychological and psychiatric problems has a long historical tradition, and far more than any other type of illness, mental disorders are subject to negative judgements [23]. Fear and discrimination of people with mental disorders, who are often considered dangerous, unpredictable or unreliable, is still widespread among general population. Therefore, despite the importance of seeking help when suffering from mental illness, stigma may stop individuals from doing so. Stigma includes the perception that people hold disapproving attitudes or behaviours towards persons with mental ill health (perceived stigma), and the self-stigma that occurs when these perceptions are internalised [24].

Stigma around seeking treatment has also been often described as self-stigma and perceived stigma. More specifically, self-stigma around seeking treatment refers to the individual’s self-devaluation or reduction in self-esteem caused by the internalization of public stigma (i.e., the belief that seeking help is a sign of weakness) [25]. Self-stigma has been found to be associated with specific coping strategies, leading to a limited use of instrumental support (e.g., getting advice or help from others) and to greater self-blame [24]. Perceived stigma around seeking treatment refers to the perception that individuals in a social group would view someone who seeks psychological treatment as being less socially acceptable [26], and it has been found to be associated with avoidance coping strategies [27].

Several studies associated stigma with overall psychological distress [28] and with negative attitudes or intentions towards seeking psychological help [29–31], also among college students [32, 33]. Lannin and colleagues [34] suggest that self-stigma may hinder initial decisions to seek mental health and counselling information, and other recent studies found that self-stigma was negatively related to attitudes towards seeking professional psychological help and help seeking behaviour [35, 36]. Jennings and colleagues [32] found that perceived stigma may influence help seeking intentions among college students by potentially increasing their stigmatizing attitudes towards themselves, while other studies found no significant associations between perceived stigma and help seeking behaviour [37, 38]. Vogel and colleagues [26] found that perceived stigma contributed to the experience of self-stigma, which, in turn, influenced help-seeking attitudes and eventually help-seeking willingness. However, the magnitudes of the relationships between perceived stigma, self-stigma and help-seeking attitudes were significantly different in samples of college students from different countries [39]. A recent meta-analysis estimating the impact of stigma on active help seeking found mixed results [40]. Hence, further studies are needed to better understand the impact of stigma on help seeking intentions and behaviour.

These findings suggest that help seeking intentions in college students may be affected by a wide range of factors. However, the studies conducted so far are inconclusive and there is no clear consensus on factors that affect help seeking intentions in college students [15, 41]. A multidimensional approach is therefore needed to understand the complexity of this phenomenon; however, there is a lack of studies analyzing the multifaceted interplay between all the different factors involved, and how they may contribute towards help seeking intentions. Experimental research is also needed to determine whether intention to seek help could be increased with the extent to which intervention strategies need to be tailored to particular student characteristics [15]. The purpose of this study was to shed light on the intentions of a considerably large sample of Italian university students to seek professional psychological help, allowing us to overcome the previous debatable findings and provide universities and health services with robust results regarding the actual relationships involved in help seeking. Specifically, we aimed to analyze how interrelationships between specific domains, such as psychological distress, perceived and self-stigma around seeking help, and coping strategies, relate to each other and to professional help seeking intentions. Based on available literature [19–22, 24, 28, 32, 35, 36], mutual associations among distress, stigma, coping strategies and help seeking intentions were all plausible, yet we expected that some relationships may arise as being predominant from the data. In particular, we hypothesized that psychological distress may affect coping strategies and/or stigma which, in turn, may modify help seeking intentions. Through the Structural Equation Models approach we verified the plausibility of these hypotheses and prior knowledge with the gathered data in a pure confirmatory framework [42, 43]. Identifying actual relationships among these factors would allow practitioners, and especially college counsellors, to identify specific areas of intervention for promoting help seeking intentions.

## Methods

### Study design and participants

This cross-sectional observational study was conducted at a medium-sized Italian university. Recruitment and data collection took place between May and June 2019, in the context of a multidimensional online survey into the psychological well-being of students. Through the list of the academic e-mail addresses provided to students and in collaboration with the University Secretariat, every enrolled student was invited to take part in the survey via e-mail. Together with the link to access the survey, the invitation included a detailed description of the study, as well as information on their voluntary participation and on the anonymity of data. Via the web link, students were asked to confirm their consent to participate. The study was conducted in accordance with the World Medical Association's Helsinki Declaration for Human Studies, while organizational approvals were obtained from the university institutional review board (approved with provision no. 56 on 27/09/2018 and then ratified in memorandum no. 11.1/2018 on 10/10/2018) before contacting students. In order to maximize the response rate, we used some of the strategies proposed by Edwards and colleagues [44], such as user-friendly questions, close-ended options for answers and sending reminders (every week, for a total of six weeks, the software sent an email to students who had not completed the survey, reminding them to participate).

### Instruments

The online survey assessed several aspects of students’ life and experiences, such as socio-demographic characteristics, academic career, and physical and mental health. More specifically, this first section included questions on students' personal life, their housing situation and daily routine, any family history of somatic and mental disorders, substance use and any current medical prescription. It also included standardized instruments for a multidimensional evaluation, including instruments for the assessment of psychological distress, coping strategies,stigma, and help seeking intentions. The following instruments are described in the same order as they were presented to the students in the online survey.

Psychological distress was measured with the University Stress Scale [45] (USS; Cronbach’s alpha = 0.83, test–retest reliability *r* = 0.82). The USS is a 21-item screening test measuring the cognitive appraisal of demands across the range of environmental stressors experienced by students. The question included in the instructions asks: *"How often have each of the following caused you stress over the past month?"* and two example items are "*Procrastination"* and *"Parental Expectations"*. Each item scores from 0 (*Not at all*) to 3 (*Constantly*), and the sum of all items gives the extent score, ranging from 0 to 63. An extent score equal to or above 13 (normed on a sample of 2,596 Australian university students) is predictive of significant psychological distress, and indicates students most likely to be experiencing depression and anxiety symptoms as a result of excessive stress [46]. In our sample, the USS demonstrated a high level of reliability, as measured by a Cronbach’s alpha value of 0.82.

Coping strategies were assessed using the Brief Coping Orientation to Problems Experienced Inventory (Brief-COPE) [47]. The Brief-COPE is a 28-item self-report questionnaire designed to measure effective and ineffective ways of coping with a stressful life event. Each item is rated on a 4-point Likert scale ranging from 1 (*I have not been doing this at all*) to 4 (*I have been doing this a lot*). It includes 14 two-item subscales, each reflecting the use of a coping strategy, which have also been grouped into three broader subscales [48, 49]: problem-focused coping strategies (including active coping, instrumental support and planning subscales, and with a total score ranging from 6 to 24), emotion-focused coping strategies (including acceptance, emotional support, humour, positive reframing and religion subscales, and with a total score ranging from 10 to 40), and avoidance/dysfunctional coping strategies (including behavioural disengagement, denial, self-distraction, self-blame, substance use and venting subscales, and with a total score ranging from 12 to 48). Some example items are: "*I've been thinking hard about what steps to take*", "*I've been learning to live with it*", and "*I've been giving up the attempt to cope*". The Brief-COPE is a validated instrument in which Cronbach’s alpha values range from 0.50 to 0.90, with only three coping strategies falling below 0.60. It has been widely used in Italian samples, showing good reliability (e.g. Chiavarino et al. [50], Cronbach's alpha ranging from 0.70 to 0.78; and Scrignaro et al. [51], Cronbach's alpha ranging from 0.54 to 0.85). In our sample it had a high reliability, with Cronbach’s alpha at 0.83 (with subscales' alpha values ranging from 0.65 to 0.76).

Self-stigma around seeking help was assessed using the Self-Stigma of Seeking Help scale [25] (SSOSH; Cronbach's alpha = 0.90, test retest reliability *r* = 0.72), a 10-item instrument that measures respondents’ level of comfort or concern in relation to seeking psychological help for a mental health problem. Items are rated from 1 (*Strongly disagree*) to 5 (*Strongly agree*), with higher total scores (ranging from 10 to 50) indicating greater self-stigma. Some example items are: "*I would feel inadequate if I went to a therapist for psychological help*" and "*It would make me feel inferior to ask a therapist for help*". In our sample, the SSOSH demonstrated high reliability (Cronbach’s alpha = 0.84).

Perceived stigma around seeking help was measured using the Perceptions of Stigmatization by Others for Seeking Help scale [52] (PSOSH; Cronbach's alpha = 0.89, test–retest reliability *r* = 0.82). The question included in the instructions states: "*Imagine you had an emotional or personal issue that you could not solve on your own. If you sought counselling services for this issue, to what degree do you believe that the people you interact with would…*" and then participants are asked to rate five items on a 5-point Likert scale (Example given: "*React negatively to you*", "*Think bad things of you*"), ranging from 1 (*Not at all*) to 5 (*A great deal*), with higher total scores (ranging from five to 25) reflecting greater perceived stigmatization by one’s social network. In our sample, the PSOSH demonstrated high reliability, with Cronbach's alpha at 0.87.

Professional help seeking intentions were assessed using the General Help Seeking Questionnaire (GHSQ) [53]. The GHSQ uses a matrix format that can be modified according to purpose and need, asking respondents to rate the likelihood of seeking help from different sources (formal -e.g. Mental Health Professional or Doctor/General Practitioner-, and informal- e.g. friend or parent-), with values ranging from 1 (*Extremely unlikely*) to 7 (*Extremely likely*). This instrument comprises two subscales, which refer to the motives for seeking help: i) emotional or personal problems; and ii) suicidal thoughts. The GHSQ demonstrated good reliability, with Cronbach’s alpha = 0.70 and test–retest reliability *r* = . 86 for emotional-personal problems, and Cronbach’s alpha = 0.83 and test–retest reliability *r* = 0.88 for suicidal thoughts. In an Italian study on high school students, D'Avanzo and colleagues [54] used a slightly modified version of the scale: test–retest reliability values ranged from 0.47 to 0.74, while Cronbach’s alpha values ranged from 0.56 to 0.69. In our sample, the overall Cronbach’s alpha value was confirmed at 0.76 (Cronbach’s alpha = 0.68 for emotional-personal problems, and Cronbach’s alpha = 0.72 for suicidal thoughts). In order to evaluate the intention of seeking professional help, in the present study, we considered responses to the items regarding the likelihood of seeking help from a mental health professional in the case of both emotional/personal problems and suicidal thoughts.

The study was conducted in Italian; therefore, we used an adapted and translated Italian version of the original scales. With regard to the USS and the GHSQ, there was not an Italian version of such scales. So, they were linguistically adapted and translated into Italian by two authors of this research team, both with fluent English skills. These two translations were synoptically compared in order to overcome any divergent translations and to achieve a full consensus. Then, a native English-speaking professional translator with fluent Italian skills was asked to translate the Italian version of the scales back into English. The authors performed the review of this secondary English translation and any incongruities were rationalized and cleared up. A final Italian version of the instruments was then formulated, checking for minor errors which have been missed during the translation process. With regard to the SSOSH, we used the Italian version available on the IOWA State University Website, where the original and translated versions of the scale are available. We contacted the author who originally translated the SSOSH, and he provided us with the Italian version of the PSOSH as well. Lastly, being the Brief-COPE very widely used also in Italian research on coping strategies, an Italian version was already available [55].

### Data analysis

Descriptive statistics for socio-demographic and academic characteristics and for the questionnaire scores were computed by percentage distribution for categorical variables, and mean and Standard Deviation (*SD*) for quantitative variables. Association analyses between individual socio-demographic/academic variables (gender) and the outcomes of instruments were examined using the Student's T-test or a corresponding non-parametric test such as Mann–Whitney (for non-Gaussian-distributed data). In detail, gaussianity assumptions for the instrument scores were evaluated by Kolmogorov–Smirnov and Shapiro–Wilk tests; for score distributions which differed from normality, the non-parametric Mann–Whitney test for group comparison was applied.

Relationships among instruments were evaluated using Spearman’s correlations. In addition, partial correlations were performed in order to evaluate the effect of socio-demographic variables (appropriately dichotomized in 0 versus 1 in the case of categorical variables) on mutual relationships.

Based on correlation analysis and specific prior literature-based [19–22, 24, 28, 32, 35, 36] hypotheses, a theoretical model representing the interrelations between psychological distress, coping strategies and stigma with the target outcome (help seeking intentions) was tested using Structural Equation Models (SEM). We followed the Bollen and Pearl framework [43], based on which the SEM method represents and relies upon the causal assumptions of the researcher, thus different models (all equally as plausible) were tested and the best was chosen as the one more plausible with the data. The main advantage of using SEM is the flexibility to model complex relationships between one or more independent (exogenous) variables and one or more dependent (endogenous) variables. Moreover, considering the different subscales of Brief Cope, the SSOSH and PSOSH scales measuring stigma, and the items of GHSQ regarding professional help seeking, we took advantage of the possibility to use SEM involving the above mentioned measures to provide latent constructs regarding coping strategies, stigma of seeking help and professional help seeking intentions. In other words, the SEM approach allowed to perform both multiple linear models (through that, relations among variables were assessed) and, simultaneously, a factor analysis for observed variables that were supposed to be associated. Through SEM, thus, we were able to analyze both: i) the relationship of the latent variables (hereafter called 'latent constructs', derived by the factor analysis), with the other variables in the model; and ii) the relationship of the single indicator variables (observed variables that contributed to the latent construct) with the other variables and latent constructs in the model, by indirect effects. In this regard, the contribution of each observed variable to the corresponding latent construct was therefore provided. SEM results were presented in terms of a standardized total, with direct and indirect effects, allowing us to investigate both relations among latent constructs and relations between latent constructs and single observed variables (indicator variables).

To see if the hypothesized model was a plausible explanatory model for the empirical data, several goodness-of-fit indices was used (https://www.sussex.ac.uk/its/pdfs/SPSS_Amos_User_Guide_22.pdf): *χ2* test (a *p*value larger than 0.05 indicates that the hypothesized model is coherent with the data), relative *χ2* test (less than 2.5 indicates a good fit), Comparative Fit Index (CFI, indicating the % of covariation in data reproduced by the model, larger than 0.95 indicates good model), Root Mean Square Error of Approximation (RMSEA, value of < 0.05 indicates good fit), Tucker–Lewis coefficient (TLI, higher than 0.9 indicates a good fit), and Akaike Information Criterion (AIC, absolute fit index, the lower value, the better the fit). The best fitted model would be the one that passes all the thresholds of the goodness of fit indices. Missing data were handled through multiple Bayesian imputation. To better represent the structure of the data (with potential correlated error among measures) and to improve the model fit, error covariance parameters were added in the SEMs according to the ‘modification index’ procedure available in the AMOS package of SPSS software. All tests were two-tailed and the probability of a type I error was set at *p* < 0.05. The analyses were performed using IBM SPSS Statistics for Windows, Version 26.0. (Armonk, NY: IBM Corp). The SEM method was implemented using the SPSS package AMOS 26.0.

## Results

Out of the 13,886 students in the study population, 3754 (27.1%) agreed to participate by accessing the online survey. The mean age of participants was 23.09 years (SD = 4.69, median value = 22, interquartile range = 20–23) and most students were female (58%) and Italian (94.8%). The majority of students were full-time students (79%), registered on a regular academic year (80.2%), attending a Bachelor's degree course (59.6%) and undergraduate (89.4%). We tested for possible differences in professional help seeking intentions (using the GHSQ) between graduate and undergraduate subsamples, and no statistical significance was found. Additional sample characteristics are reported in Table 1.

**Table 1:** Characteristics of the sample

	N	%
*Gender*		
Male	1569	41.8
Female	2178	58.0
Other	7	0.2
Total	3754	100.0
*Citizenship*		
Italian	3556	94.8
Other EU country	99	2.7
Other non-EU country	95	2.5
Total	3750	100.0
*Attended Degree*		
Bachelor's Degree	2061	59.6
Master's Degree	366	10.6
Combined Bachelor's and Master's Degrees^a^	1032	29.8
Total	3459	100.0
*University status*		
Full time student	2730	79.0
Part-time student	727	21.0
Total	3457	100.0
*Living Status*		
In-town students	2171	62.8
Students living away from home	1287	37.2
Total	3458	100.0
*Field of study*		
Medicine	1330	38.5
Engineering	1069	30.9
Economics	754	21.8
Law	306	8.8
Total	3459	100.0
*Registration*		
Registered on a regular academic year	2774	80.2
Registered on a supplementary year	684	19.8
Total	3458	100.0

^a^These degree programs provide a Master’s degree after a single cycle of 5 or 6 years in various disciplines (e.g. Medicine, Law)

Total mean scores and gender differences for all instruments are provided in Table 2. Table 3 shows correlations among the administered instruments.

**Table 2:** Total mean scores, by gender

Instruments scores	Total	Male	Female	Test statistics	*p*
	Mean (SD)	Mean (SD)	Mean (SD)		
USS	14.45 (7.70)	13.53 (7.64)	15.1 (7.66)	*t* = −5.42	< .001
SSOSH	25.71 (6.9)	27.24 (7.10)	24.61 (6.52)	*t* = 9.75	< .001
PSOSH	8.23 (3.61)	8.50 (3.70)	8.01 (3.48)	*U*st = −4.01	< .001^a^
GHSQ—emotional or personal problems	3.41 (1.72)	3.12 (1.70)	3.61 (1.70)	*U*st = 7.40	< .001^a^
GHSQ—suicidal thoughts	4.50 (2.17)	4.30 (2.20)	4.64 (2.14)	*U*st = 4.04	< .001^a^
Brief-COPE—problem focused strategies	16.09 (3.38)	15.71 (3.45)	16.37 (3.31)	*t* = −5.17	< .001
Brief-COPE—emotion focused strategies	21.93 (4.48)	21.56 (4.54)	22.20 (4.42)	*t* = −3.79	< .001
Brief-COPE—Avoidant/dysfunctional strategies	23.48 (4.37)	22.83 (4.42)	23.93 (4.26)	*t* = −6.70	< .001

USS: University Stress Scale; SSOSH: Self-Stigma of Seeking Help scale; PSOSH: Perceptions of Stigmatization by Others for Seeking Help scale; GHSQ: General Help seeking Questionnaire; Brief-COPE: Brief Coping Orientation to Problems Experienced inventory; *t*: statistic *t* of the *t*-test; *U*st: standardized Mann–Whitney *U* statistic

^a^Non-parametric Mann–Whitney test applied for non-Gaussian distributed data: *p* values of Kolmogorov–Smirnov and Shapiro–Wilk tests both < 0.05

**Table 3:** Spearman’s correlations for instruments

Variable	1	2	3	4	5	6	7	8
1. GHSQ—Suicide thoughts	–							
2. GHSQ—Emotional or personal problems	0.46^a***^	–						
3. PSOSH	–0.14^***^	–0.07^***^	–					
4. SSOSH	–0.40^a***^	–0.48^a***^	0.30^a***^	–				
5. Brief Cope: Emotion focused strategies	0.15^***^	0.10^***^	–0.03	–0.15^***^	–			
6. Brief Cope: Problem focused strategies	0.20^a***^	0.11^***^	–0.14^***^	–0.22^a***^	0.65^a***^	–		
7. Brief Cope: Avoidance dysfunctional strategies	0.05	0.11^***^	0.23^a***^	0.02	0.33^a***^	0.13^***^	–	
8. USS	–0.02	0.09^***^	0.29^a***^	0.01	0.07^***^	–0.04	0.44^a***^	**–**

Due to the large sample size, all the coefficients larger than 0.10 were all significantly different from zero

USS: University Stress Scale; SSOSH: Self-Stigma of Seeking Help scale; PSOSH: Perceptions of Stigmatization by Others for Seeking Help scale; GHSQ: General Help seeking Questionnaire; Brief-COPE: Brief Coping Orientation to Problems Experienced inventory

^a^We consider as relevant the correlations with coefficients larger than 0.20

***p < .001

**Fig. 1:**
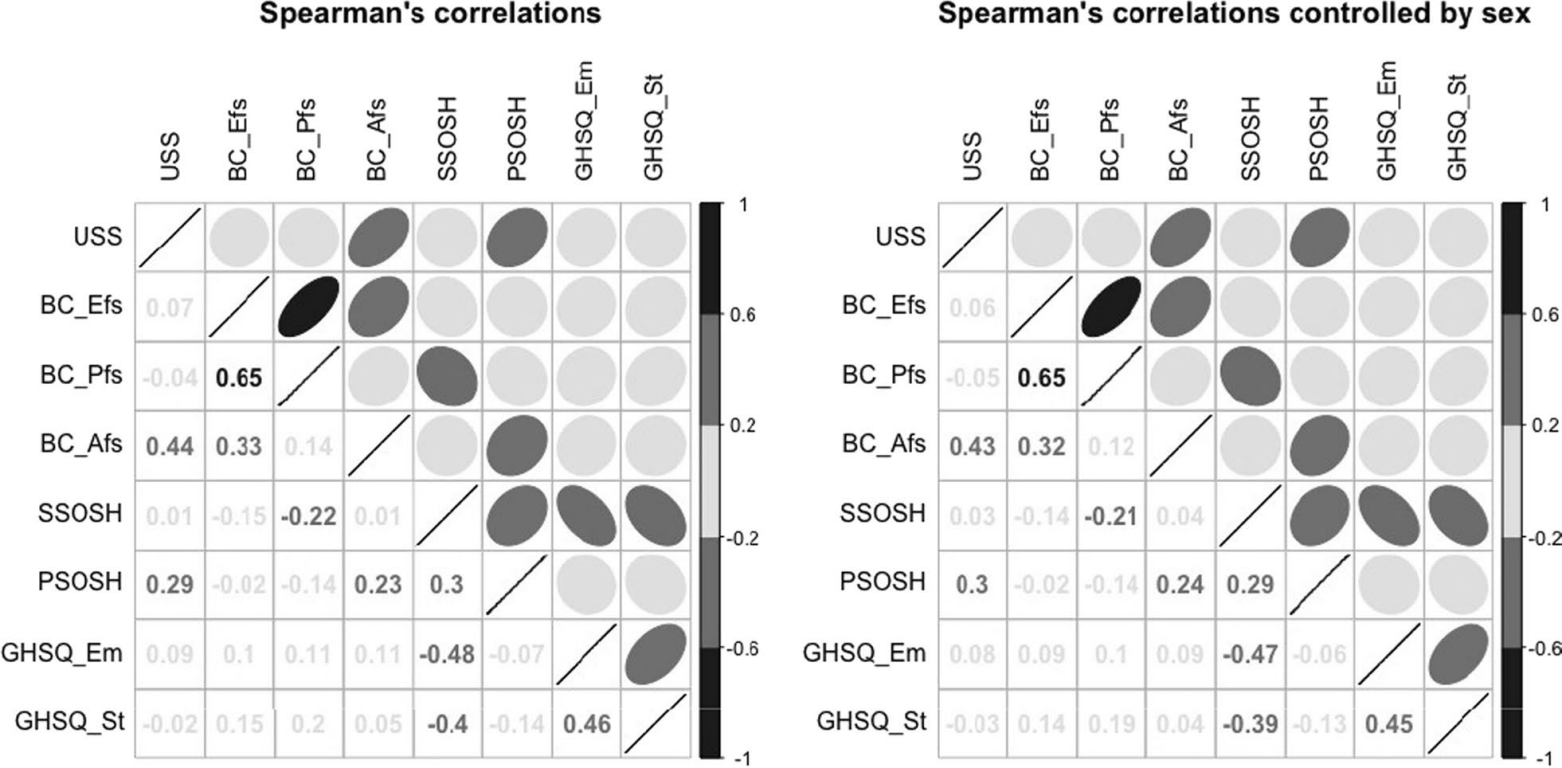
Spearman’s correlations (on the left) and partial correlations, controlled by sex (on the right). *Note.* USS: University Stress Scale score; BC_Efs: Brief-COPE- Emotion focused strategies; BC_Pfs: Brief-COPE—Problem focused strategies; BC_Afs: Brief-COPE—Avoidant/dysfunctional Strategies; SSOSH: Self-Stigma of Seeking Help scale score; PSOSH: Perceived Stigma of Seeking Help scale score; GHSQ_Em: GHSQ-likelihood of seeking help from a mental health professional for: emotional or personal problems; GHSQ_St: GHSQ-likelihood of seeking help from a mental health professional for: suicide thoughts

The highest correlation coefficients were observed between Self-stigma of seeking help and Intentions of seeking professional help for suicidal thoughts (medium-large negative correlation, *r* = −0.396, *p* < .001) and for Emotional/personal problems (moderate-large negative correlation, *r* = −0.484, *p* < .001). A moderate-large positive correlation (*r* = 0.439, *p* < .001) was observed between Avoidance/dysfunctional coping strategies and Psychological distress. Smaller correlations (small-medium positive relations) were found between Perceived stigma of seeking help and Avoidance/dysfunctional coping strategies (*r* = 0.231, *p* < 0.001) and between Problem-focused strategies and Intention of seeking professional help for suicidal thoughts (*r* = 0.197, *p* < 0.001). A negative small-medium correlation was found, on the other hand, between Self-stigma of seeking help and Problem-focused coping strategies (*r* = -0.219, *p* < 0.001). Unexpectedly, the only other variable moderately related to the USS score was Perceived stigma of seeking help (*r* = 0.287, *p* < 0.001). The strength of these correlations remained substantially unchanged even after checking for a gender effect. Correlation and partial correlation plots are reported in Fig. 1.

**Fig. 2:**
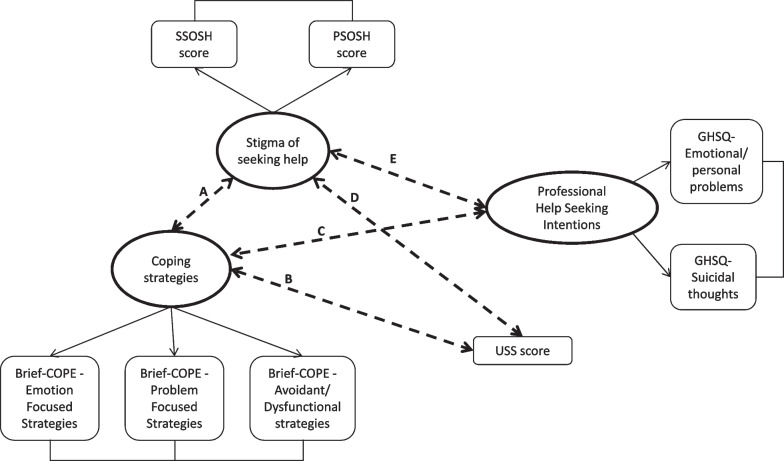
Hypothesized Structural Equation Model based on the correlation analysis. *Note*. The ellipses represent the latent constructs; the rectangles represent the observed variables (or indicator when related to a latent construct). Dashed lines indicate the theoretical relationships to be tested and validated by data through the Structural Equation Model approach. Based on correlation analysis results, the mutual associations (A-E) between distress, stigma, coping strategies and help seeking intentions were all plausible. **A** relationship mainly due to PSOSH score related with Brief-COPE Avoidance/Dysfunctional St., and to SSOSH related with Brief-COPE Problem-Focused St.; **B** relationship mainly due to USS score related with Brief-COPE Avoidance/Dysfunctional St.; **C** relationship mainly due to Brief-COPE Problem-Focused St. related with GHSQ –Seeking professional help for suicidal thoughts; **D** relationship mainly due to USS score related with PSOSH score; **E** relationship mainly due to SSOSH score related with GHSQ–Seeking professional help for suicidal thoughts and emotional/personal problems.

Based on the measurements' correlations found in our sample and on the relevant literature [19–22, 24, 28, 32, 35, 36], a theoretical model representing the structure of the relationships between measures was hypothesized (Fig. 2). From this, specific relationships between variables were investigated through several fitted SEMs. In detail, psychological distress (measured with the USS) was hypothesized to be related to both the Coping Strategies latent construct and the Stigma of Seeking Help latent construct; conversely, these latent constructs were assumed to be related to each other and to the Professional Help seeking Intentions latent construct.

Previous findings [19–22, 24, 28, 32, 35, 36] had given no conclusive indications regarding the direction of the associations among the single observed psychological scales, nor among the more general domains (coping, stigma and help seeking) included in the hypothesized model (almost all associations substantially appear plausible). Therefore, several SEMs were used in order to discover the most relevant and plausible relationships for our large sample of students (see the SEMs tested in the Additional file 1).

**Fig. 3:**
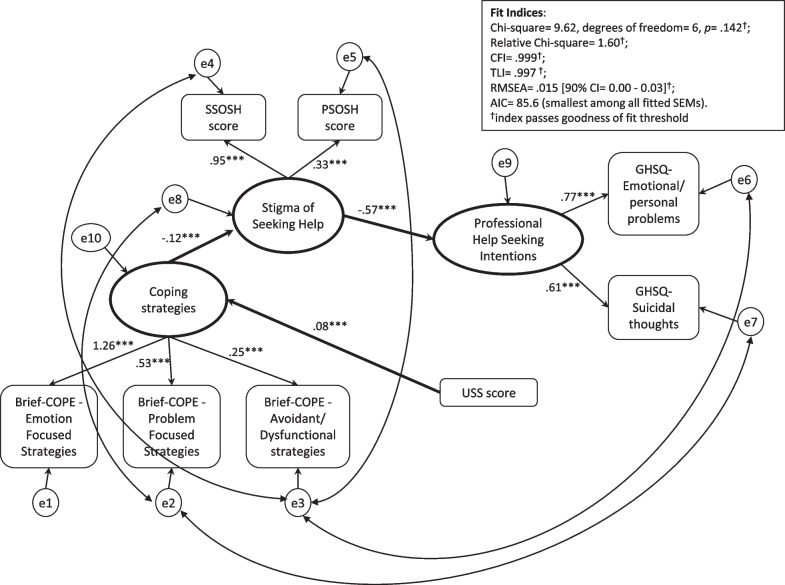
Best-fit Structural Equation Model. *Note*. The small circles represent the error of the observed (endogenous) variables. The arches represent the error covariances (set to improve the fit). Standardized regression weights (or factor loading when referring to indicators of latent constructs) are reported above the arrows. All the regression weights (standardized beta coefficients) are significant at *p* < .001. Model fit indices*:χ*^2^ = 9.62, degree of freedom = 6, *p* = .142; relative χ2 = 1.60, CFI = .999; TLI = .997, RSMEA = .015 [90% Confidence Interval .00 – .03], AIC = 85.6. ^***^p < .001

The best-fit SEM is reported in Fig. 3. Model fit indices showed a good fit of the data (*χ*^2^ = 9.62, degree of freedom = 6, *p* = 0.142; relative *χ*2 = 1.60, CFI = 0.999; TLI = 0.997, RSMEA = 0.015 [90% Confidence Interval 0.00–0.03], and AIC = 85.6).

Latent constructs (computed de facto by a factor analysis on the observed indicator variables) were well defined by their indicators. The main contributions to the Coping Strategies latent construct were given by Brief-COPE Emotion- and Problem-focused strategies (factor loading = 1.26 and 0.53 respectively), while the main contribution to the Stigma of Seeking Help latent construct was given by the SSOSH score (factor loading equal to 0.95), confirming the relevance of SSOSH with respect to PSOSH (factor loading = 0.33) in the evaluation of Stigma of Seeking Help. The Professional Help seeking Intentions latent construct was defined with a substantially equal contribution of its indicators: GHSQ-likelihood of seeking help from a mental health professional for suicidal thoughts (factor loading = 0.77) and GHSQ-likelihood of seeking help from a mental health professional for emotional or personal problems (factor loading = 0.61). According to the best-fit SEM, data supported the following chain of relations (see Fig. 3 and Table 4): the USS score was directly and positively related to the Coping Strategies latent construct (standardized regression weights *β* = 0.08, *p* < 0.001), which in turn was negatively related to the Stigma of Seeking Help latent construct (*β* = −0.12, *p* < 0.001). Lastly, the Stigma of Seeking Help latent construct was negatively related to the Professional Help seeking Intentions latent construct (*β* = −0.67, *p* < 0.001). In addition, Professional Help seeking Intentions were indirectly and positively related to the USS score and Coping Strategies latent construct(*β* = −0.01, *p* < 0.001 and *β* = 0.08, *p* < 0.001, respectively). Moreover, through the indirect effects, we found that the USS was mainly related to the Brief-COPE – Emotion-focused strategies (see Table 4), which was the scale that mainly contributed to the Coping Strategies latent construct. Similarly, we found that the USS had a larger indirect effect on the SSOSH score than on the PSOSH score.

**Table 4:** Standardized total, direct and indirect effects of the best fitted SEM

		USS	Coping Strategies	Stigma of seeking help	Professional help seeking intentions
*Latent constructs*					
Coping Strategies	Total	0.075	0.000	0.000	0.000
	Direct	0.075	0.000	0.000	0.000
	Indirect	0.000	0.000	0.000	0.000
Stigma of seeking help	Total	−0.009	−0.120	0.000	0.000
	Direct	0.000	−0.120	0.000	0.000
	Indirect	−0.009	0.000	0.000	0.000
Professional help seeking intentions	Total	0.006	0.080	−0.569	0.000
	Direct	0.000	0.000	−0.569	0.000
	Indirect	0.006	0.080	0.000	0.000
*Observed variables*					
GHSQ—suicidal thoughts	Total	0.004	0.049	−0.409	0.611
	Direct	0.000	0.000	0.000	0.611
	Indirect	0.004	0.049	−0.409	0.000
GHSQ—emotional/personal problems	Total	0.005	0.062	−0.514	0.768
	Direct	0.000	0.000	0.000	0.768
	Indirect	0.005	0.062	−0.514	0.000
PSOSH	Total	−0.003	−0.040	0.333	0.000
	Direct	0.000	0.000	0.333	0.000
	Indirect	−0.003	−0.040	0.000	0.000
SSOSH	Total	−0.009	−0.114	0.950	0.000
	Direct	0.000	0.000	0.950	0.000
	Indirect	−0.009	−0.114	0.000	0.000
Brief-COPE—emotion Focused Strategies	Total	0.095	10.261	0.000	0.000
	Direct	0.000	10.261	0.000	0.000
	Indirect	0.095	0.000	0.000	0.000
Brief-COPE—problem Focused Strategies	Total	0.040	0.534	0.000	0.000
	Direct	0.000	0.534	0.000	0.000
	Indirect	0.040	0.000	0.000	0.000
Brief-COPE—avoidance/dysfunctional Strategies	Total	0.019	0.248	0.000	0.000
	Direct	0.000	0.248	0.000	0.000
	Indirect	0.019	0.000	0.000	0.000

The reported values are the regression weights (regression beta coefficients). All coefficients are statistically significant at level *p* < 0.001). Total effects are the effects of a variable (observed or latent) on another variable through the path defined by the model. Total effect is the sum of the direct (path where a direct arrow exists) and indirect (path defined through more than one arrow) effects

USS: University Stress Scale; SSOSH: Self-Stigma of Seeking Help scale; PSOSH: Perceptions of Stigmatization by Others for Seeking Help scale; GHSQ: General Help seeking Questionnaire; Brief-COPE: Brief Coping Orientation to Problems Experienced inventory

Considering the other tested SEMs, and therefore the other relationships between variables that were tested, results suggest that the data did not support the following relationships: i) Stigma of Seeking Help as a mediator of the relationship between Coping Strategies and Professional Help seeking Intentions (*χ2* = 16.48; degree of freedom = 5; *p* = 0.006; relative *χ2* = 3.30; CFI = 0.95; TLI = 0.98; RSMEA = 0.03 [90% Confidence Interval 0.01–0.05]; and AIC = 94.5, see Additional file 1, Fig. S1); ii) the direct relationship between Coping Strategies and Professional Help seeking Intentions (*χ2* = 40.75; degree of freedom = 4; *p* < 0.001; relative *χ2* = 10.19; CFI = 0.90; TLI = 0.95; RSMEA = 0.06 [90% Confidence Interval 0.04–0.08]; and AIC = 120.8, see Additional file 1, Fig. S2); iii) the USS score as an endogenous (dependent) variable related to the Coping Strategies (exogenous) variable (*χ2* = 38.1; degree of freedom = 5; *p* < 0.001; relative *χ2* = 7.6; CFI = 0.95; TLI = 0.96; RSMEA = 0.05 [90% Confidence Interval 0.04–0.07]; and AIC = 116.1, see Additional file 1, Fig. S3).

## Discussion

Mental health issues are common among university students, but they are quite reluctant to seek professional psychological help when they need it. Researchers suggest that help seeking intentions in college students may be affected by a wide range of factors, including stigma, coping strategies and psychological distress [19–22, 24, 28, 32, 35, 36]. However, previous findings did not provide conclusive indications regarding the multifaceted interplay between these factors and there is a need for a multidimensional approach to understand the complexity of this phenomenon. The main purpose of this study was therefore to determine the role of psychological distress, stigma and coping strategies on intentions to seek professional help for psychological problems in a large sample of Italian university students, in order to overcome the previous debatable findings and provide universities and health services with robust results.

In our sample, the mean GHSQ scores indicated that it was rather "unlikely" that students would seek help from a mental health professional for emotional or personal problems, and that this was slightly more "likely" in the case of suicidal thoughts. This highlights the impact of the stigma and reluctance associated with mental health services among university students, especially when the level of distress is high. Indeed, the majority of students scored above the cut-off on the USS scale, indicating significant levels of psychological distress, thus confirming several epidemiological studies in which young people, including college students, were classed as a vulnerable population in terms of the onset of mental health issues [56, 57].

Women showed higher levels of distress compared to men, and this is also in line with findings reported in literature [58, 59]. Another interesting gender difference regarded stigma: male students showed higher levels of both self-stigma and perceived stigma around seeking professional help. This difference confirms previous research [25, 60] and may reflect social stereotypes whereby men are expected to be stoic, controlled and self-sufficient [61], and are therefore considered weak if they ask for help or admit they are unable to handle their problems on their own. In our sample, a significant difference between male and female students was found in all scales; however, the strength of the correlations remained substantially unchanged even after controlling for the effect of gender. This means that, although male and female students showed different patterns in terms of psychological distress, coping strategies, stigma and intentions to seek professional help, the interrelations among these domains were similar for both genders.

In order to highlight these interrelations, we tested different SEMs. The best-fit model showed a direct and positive effect of the USS score on the Coping Strategies latent construct, which was in turn negatively related to the Stigma of Seeking Help latent construct, which was ultimately and negatively related to the Professional Help seeking Intentions latent construct. This chain of effects suggests that students with significant psychological distress use coping strategies to face the stigma of seeking help: the lower the stigma of seeking help, the higher the chance of developing intentions to seek professional help. However, it is worth noting that the coefficients of the associations from the USS to the Coping Strategies latent construct, and from Coping Strategies latent construct to the Stigma of Seeking Help latent construct, were relatively small (due to small regression coefficients), and this might suggest a minor clinical significance. In addition, the best-fit model showed that Professional Help seeking Intentions were indirectly and positively related to the USS score and Coping Strategies latent construct. Therefore, in order to promote professional help seeking among students, it is essential to consider stigma (which had a direct effect on help seeking intentions), but also the role of coping strategies and psychological distress, which showed significant indirect effects on help seeking intentions.

It is important to note that psychological distress was mainly related to the Brief-COPE Emotion-focused strategies score (indirect effect = 0.095), which mainly contributed to the Coping Strategies latent construct. This means that high levels of psychological distress trigger specific coping strategies, including acceptance, emotional support and positive reframing. Therefore, when students feel distressed, they may prefer to look for comfort and understanding from someone, rather than trying to come up with a strategy about what to do (which is a problem-focused strategy).This is in line with a recent study conducted by Saczuk and colleagues [62] among medical students, finding that mostly emotion-focused coping strategies were chosen by students experiencing high levels of psychological stress. However, in another recent study, higher scores of psychological stress were associated with a lower frequency use of emotion-focused strategies [20]. This may be explained considering that the latter study was conducted among hypertensive patients with a mean age close to 60 years, so such a different study population may have a very different way to cope with stressors. Further studies analysing the impact of psychological distress on coping strategies may help clarify such contrasting results. Moreover, a recent review aimed at identifying coping strategies of healthcare professional students [19] reported that positive coping strategies (including problem-focused strategies) were associated with reduced levels of psychological stress.

These insights may be very useful when implementing interventions, giving specific indications on which coping strategies to promote considering the different levels of psychological distress. Interesting suggestions for the implementation of effective programs may also be found when considering that the main contribution to the Stigma of Seeking Help latent construct was given by the SSOSH score (self-stigma). The feeling of inadequacy or weakness was therefore a greater obstacle than the feeling of mistreatment or devaluation by significant others. These findings may be interpreted through the model tested by Vogel and colleagues [26], in which public stigma appeared to lead to an internalization of negative external messages as self-stigma, and self-stigma was linked to attitudes toward psychological services. Self-stigma has been recognized as the primary determinant of attitudes toward psychological services in international samples [39, 63]. Moreover, psychological distress showed a larger indirect effect on self-stigma than on perceived stigma; as a result, we can assume that self-stigma may be a greater hindrance to distressed students’ chances of developing help seeking attitudes.

### Implications for practice

Because students are often reluctant to seek professional help, they should feel safe and encouraged to contact formal mental health services, if they need them. College counselling services should therefore adopt a youth-friendly approach, including a comfortable location and an easy to access service providing a confidential, inclusive and safe space where to feel respected and valued. Students may also not be aware of the existing counselling services, thus effective initiatives aimed at promoting such services, also through social media platforms, should be considered.

In order to gain a comprehensive picture and to detect specific keys for the implementation of effective actions to promote help seeking intentions in college students, our SEM approach highlighted the importance of taking into account all factors that have both direct and indirect effects on help seeking intentions, including stigma, coping strategies, and psychological distress. With regard to stigma, interventions should be focused firstly on self-stigma and secondly on perceived stigma, taking into consideration the level of psychological distress and social stereotypes associated with psychological issues and help seeking behaviours. There is an urgent need to shift the perspective from help seeking as a sign of weakness to help seeking as a sign of strength, confidence and resourcefulness. There is a growing body of literature assessing interventions aimed to reduce self-stigma, and the most recent reviews conducted on this topic showed that such interventions are effective and produce multiple improvements, including changes in subjective recovery, coping strategies, and self-esteem [64, 65]. Psycho-education is a common element in most of the interventions aimed to reduce self-stigma [64, 65], but it is also very common in interventions targeting perceived stigma [66], and this might guide the implementation of specific interventions for college students as well. College counsellors and other mental health practitioners might need to pay specific attention in addressing stigma in male students, which reflects traditional masculinity norms that invoke some men’s power and dominance. Such norms may lead to difficulties in expressing emotions and restrictions in behaviours (such as crying) based on specific gender roles [67]. Embracing a healthier masculinity model, and therefore promoting the qualities of strength and control among male students who seek help is one of the first steps that college counsellors should take in order to promote help seeking intentions.

As we mentioned before, improving effective coping strategies is a key factor of the promotion of help seeking intentions, and psycho-educational interventions have already demonstrated their potential in improving coping skills in students [68, 69]. Programs about coping should focus on promoting emotion-focused strategies (such as the use of emotional support, acceptance and positive reframing), and problem-focused strategies (such as active coping, planning and use of instrumental support), taking into consideration the role of psychological distress in guiding the choice of such strategies. Our results showed that students with high levels of psychological distress mostly use coping strategies that are aimed at regulating their emotions; therefore, they may need help in expanding the number and type of strategies, also including problem-focused strategies which are aimed at changing the stressful situation. Indeed, a "one size fits all" approach for the promotion of help-seeking intentions is unlikely to be effective for everyone, and a preliminary assessment of the level of psychological distress may greatly contribute to the implementation of a personalized approach.

### Limitations

The current study had a few limitations. Firstly, the large sample of students who completed the survey ensured robust findings, but perhaps did not provide the best generalization. In fact, the sample was not representative on a national scale as it came from a single university in the north of Italy. Furthermore, since participation in the study was voluntary, students who chose to participate may differ from those who declined the invitation, in terms of their characteristics and intentions. Further studies including more representative samples and cross-cultural comparisons are therefore needed to improve the generalizability of results. The self-report nature of our measures may be considered as a second limitation, in terms of the validity of results; having said that, in this study, the variables of interest mainly focused on individual perceptions (i.e., intention of seeking help, stigma and psychological distress), which are therefore better captured by self-report measures. Moreover, the coefficients of the associations from the USS to the Coping Strategies latent construct, and from Coping Strategies latent construct to the Stigma of Seeking Help latent construct were relatively small, and this might suggest a minor clinical significance, despite the statistical significance. Lastly, our data were collected cross-sectionally and the associations examined in this study cannot determine causality. Further longitudinal studies are needed to better understand the relevance and the nature of such associations.

## Conclusions

The fact that young people scarcely use mental health services is well documented [70] and it is fundamental to understand which factors may encourage or dissuade students when it comes to seek professional help. Universities need to increase and strengthen mental health services for their students and implement specific interventions for the promotion of help seeking intentions (including training on effective coping strategies and programs for the reduction of psychological distress), while policy makers need to support actions aimed at educating the general population, especially young people, about mental health. Fostering a stigma-free environment is essential for planning services that are truly accessible to students, who should no longer feel afraid or ashamed to ask for the help they need.

## Supplementary Information

**Additional file 1**: Additional analysis and supplementary figures.

## Data Availability

The data that support the findings of this study are available from the corresponding author upon reasonable request.

## Abbreviations

USS: University Stress Scale
Brief-COPE: Brief Coping Orientation to Problems Experienced Inventory
SSOSH: Self-Stigma of Seeking Help scale
PSOSH: Perceptions of Stigmatization by Others for Seeking Help scale
GHSQ: General Help Seeking Questionnaire
SD: Standard Deviation
SEM: Structural Equation Model
CFI: Comparative Fit Index
RMSEA: Root Mean Square Error of Approximation
TLI: Tucker–Lewis coefficient
AIC: Akaike Information Criterion
