# Supplementary material for: The role of psychological distress, stigma and coping strategies on help-seeking intentions in a sample of Italian college students
Source: BMC Psychol. 2023 Jun 6;11:177. doi: 10.1186/s40359-023-01171-w (PMC10243082; doi:10.1186/s40359-023-01171-w)
Supplement: Supplementary file 1 — Additional file 1: Additional analysis and supplementary figures. [file 40359_2023_1171_MOESM1_ESM.pdf]

Figure S1. First Structural Equation model tested (SEM1)

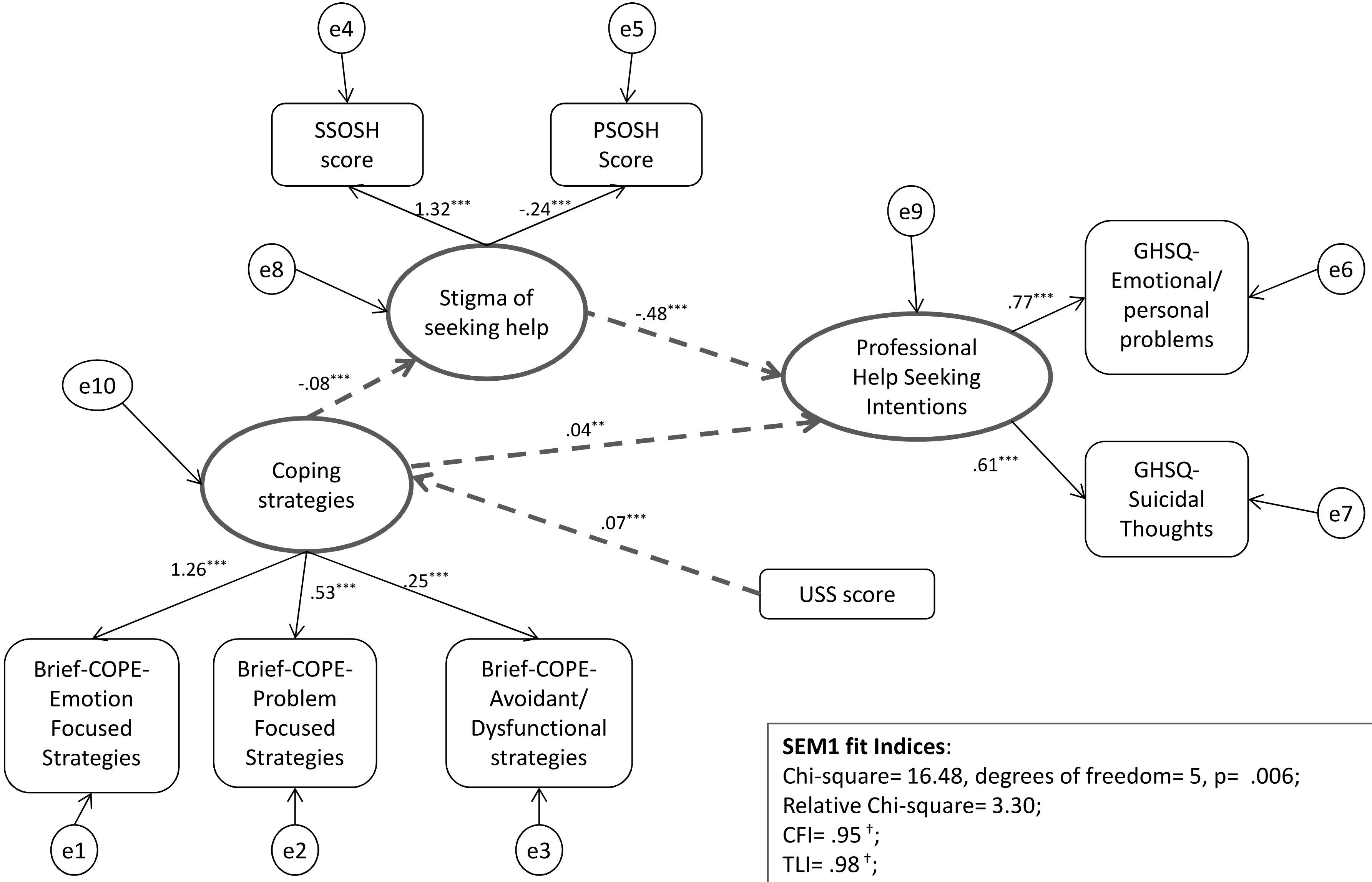

SEM1 fit Indices:
Chi-square= 16.48, degrees of freedom= 5, p= .006;
Relative Chi-square= 3.30;
CFI= .95 †;
TLI= .98 †;
RMSEA= .03 [90%CI: 0.01 -0.05]†;
AIC=94.5

† index passes goodness of fit threshold

**Regression weights (beta coefficients) significant at level *p* <.01

***Regression weights (beta coefficients) significant at level *p* <.001

Figure S2. Second Structural Equation model tested (SEM2)

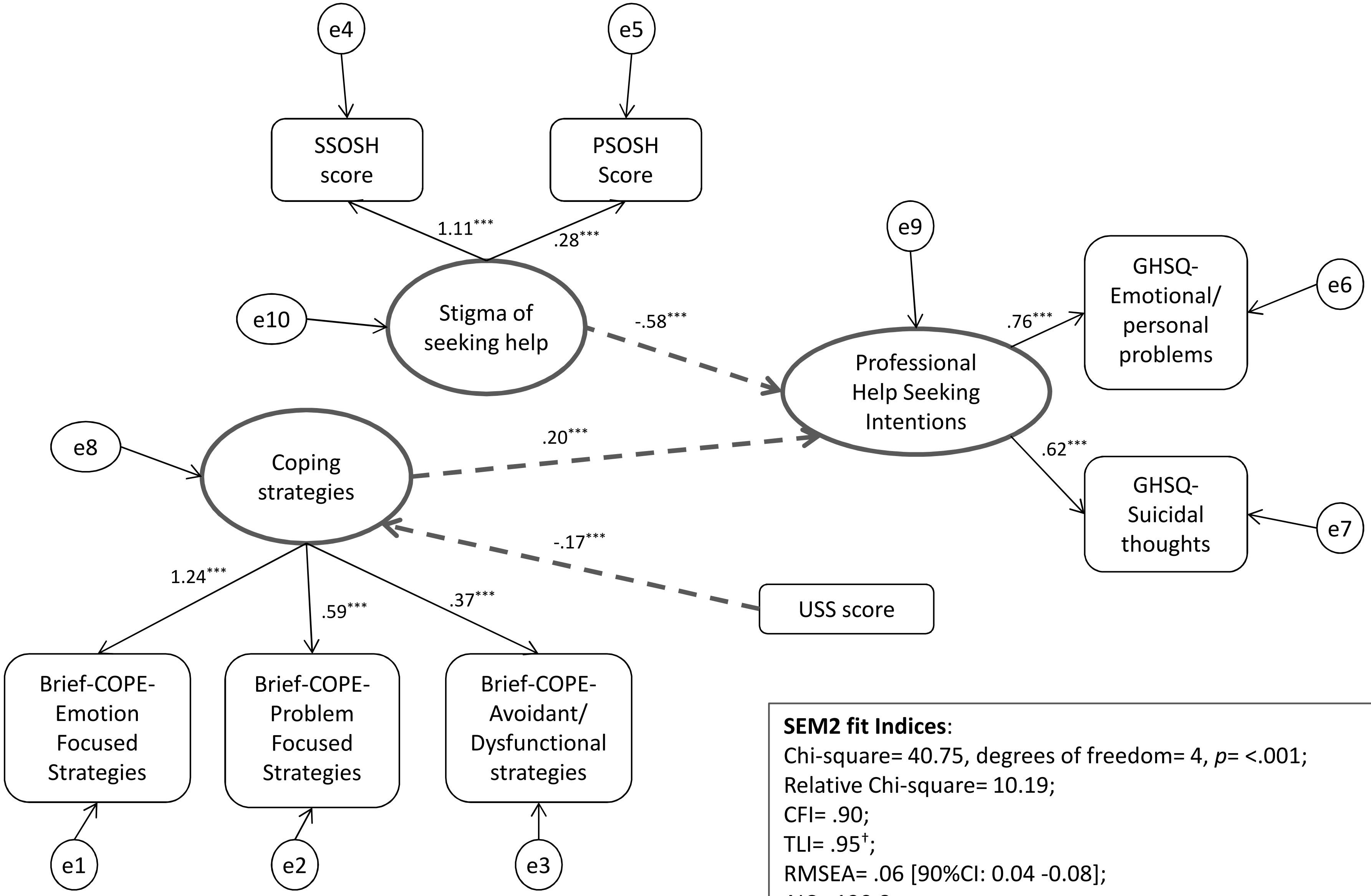

***Regression weights (beta coefficients) significant at level *p* <.001

SEM2 fit Indices:
Chi-square= 40.75, degrees of freedom= 4, *p*= <.001;
Relative Chi-square= 10.19;
CFI= .90;
TLI= .95†;
RMSEA= .06 [90%CI: 0.04 -0.08];
AIC= 120.8

† index passes goodness of fit threshold

Figure S3. Third Structural Equation model tested (SEM3)

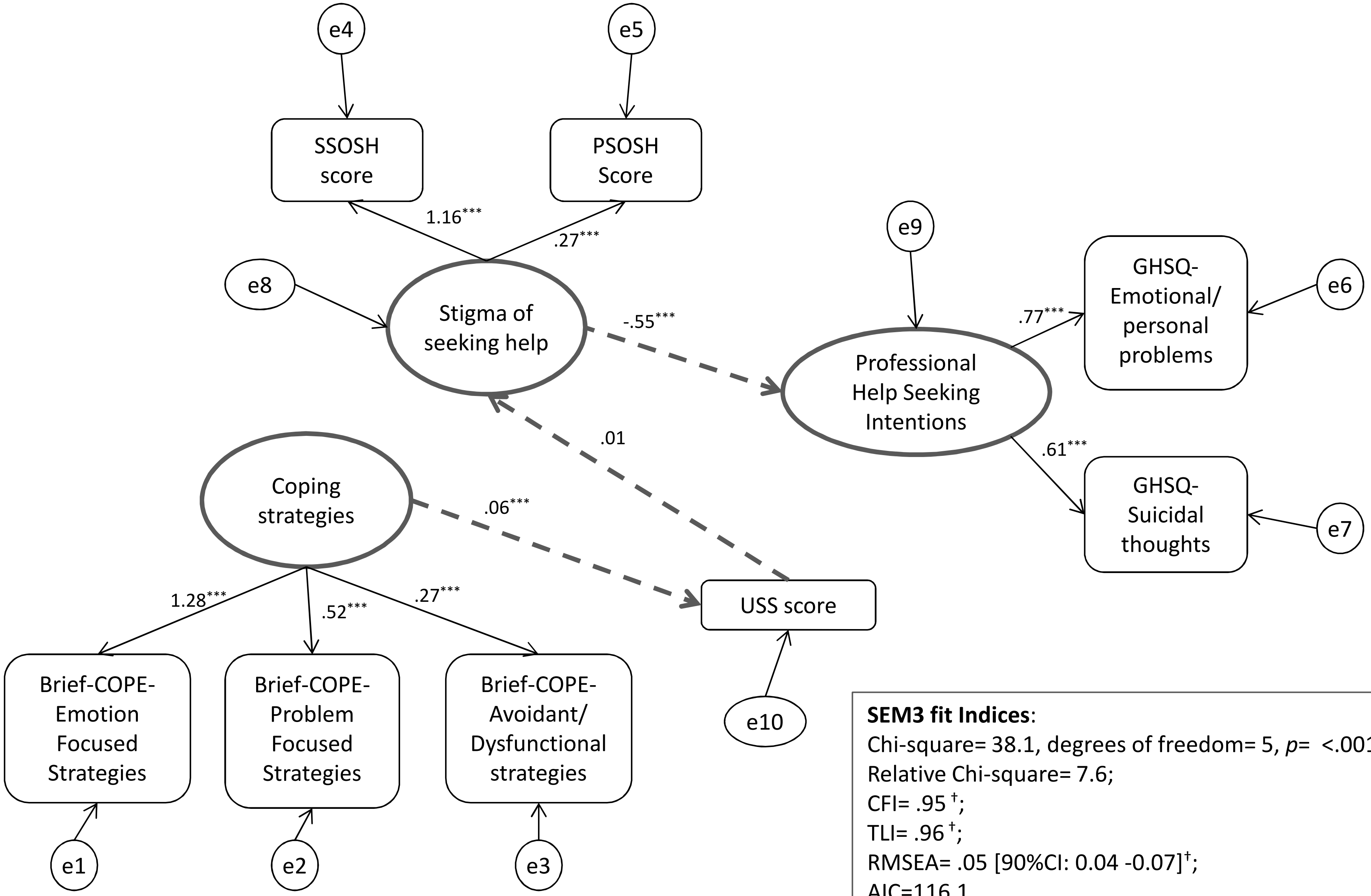

SEM3 fit Indices:
Chi-square= 38.1, degrees of freedom= 5, *p*= <.001;
Relative Chi-square= 7.6;
CFI= .95 †;
TLI= .96 †;
RMSEA= .05 [90%CI: 0.04 -0.07]†;
AIC=116.1

† index passes goodness of fit threshold

***Regression weights (beta coefficients) significant at level *p* <.001
